# Effects of Lycopene on the Initial State of Atherosclerosis in New Zealand White (NZW) Rabbits

**DOI:** 10.1371/journal.pone.0030808

**Published:** 2012-01-25

**Authors:** Mario Lorenz, Mandy Fechner, Janine Kalkowski, Kati Fröhlich, Anne Trautmann, Volker Böhm, Gerhard Liebisch, Stefan Lehneis, Gerd Schmitz, Antje Ludwig, Gert Baumann, Karl Stangl, Verena Stangl

**Affiliations:** 1 Medizinische Klinik für Kardiologie und Angiologie, Campus Mitte, Charité - Universitätsmedizin Berlin, Berlin, Germany; 2 Institute of Nutrition, Friedrich Schiller University Jena, Jena, Germany; 3 Food GmbH Jena-Analytik Consulting Jena, Jena, Germany; 4 Institut für Klinische Chemie und Laboratoriumsmedizin, Universitätsklinikum Regensburg, Regensburg, Germany; Universität Würzburg, Germany

## Abstract

**Background:**

Lycopene is the main carotenoid in tomatoes, where it is found in high concentrations. Strong epidemiological evidence suggests that lycopene may provide protection against cardiovascular diseases. We therefore studied the effects of lycopene on diet-induced increase in serum lipid levels and the initiation of atherosclerosis in New Zealand White (NZW) rabbits.

**Methodology/Principal Findings:**

The animals, divided into four groups of 9 animals each, were fed either a standard diet, a high-cholesterol diet containing 0.5% cholesterol, a high-cholesterol diet containing placebo beadlets, or a high-cholesterol diet plus 5 mg/kg body weight/day of lycopene (in the form of lycopene beadlets), for a period of 4 weeks. We found significantly elevated lycopene plasma levels in the animal group treated with lycopene beadlets. Compared to the high-cholesterol and the placebo group, this was associated with a significant reduction of 50% in total cholesterol and LDL cholesterol serum levels in the lycopene group. The amount of cholesteryl ester in the aorta was significantly decreased by lycopene. However, we did not observe a significant decrease in the extent of aortic surface lipid accumulation in the lycopene group. In addition, no differences in the intima-media thickness among groups were observed. Endothelial-dependent and endothelial-independent vasodilation in isolated rabbit aortic and carotid rings did not differ among any of the animal groups.

**Conclusions:**

Lycopene supplementation for 4 weeks increased lycopene plasma levels in the animals. Although we found strongly reduced total and LDL cholesterol serum levels as well as significantly lower amounts of cholesteryl ester in the aortae in the lycopene-treated group, no significant differences in initial lesions in the aortae were detected.

## Introduction

Epidemiological studies indicate that the consumption of tomatoes and tomato products is inversely associated with the prevalence of cardiovascular diseases [1]. Tomatoes contain a plethora of ingredients. During recent years, the carotenoid lycopene has attracted much attention for its potentially beneficial cardiovascular effects [2], [3]. Lycopene is located mainly in tomato peels and contributes to the red colour of tomatoes. A number of underlying mechanisms for protective cardiovascular actions of lycopene have been suggested in cell culture studies. These include inhibition of smooth muscle cell proliferation and foam cell formation, prevention of endothelial cell injury, modulation of cholesterol metabolism and inhibition of LDL oxidation, and decrease of pro-inflammatory cytokines [4]. Antioxidant effects of lycopene are discussed to play a key role [5]. A number of studies have shown an inverse correlation of circulating plasma lycopene levels with adverse cardiovascular parameters in humans. In the Rotterdam study, higher lycopene serum levels were modestly associated with reduced aortic calcification [6]. Elevated plasma levels of lycopene correlated with decreased intima-media thickness in the carotid artery, a parameter of early stages of atherosclerosis [7]–[9]. An inverse relationship between circulating lycopene plasma levels and arterial stiffness was observed [10], [11]. The impact of lycopene on blood cholesterol levels in human intervention studies appears to be dose-dependent [12].

Growing evidence from in vitro experiments as well as from epidemiological and intervention studies on the cardiovascular beneficial effects of lycopene prompted us to investigate the impact of lycopene supplementation on diet-induced increase in serum lipid levels and the initiation of atherosclerosis in New Zealand White (NZW) rabbits. Rabbits have been widely used as an animal model for the study of atherosclerosis [13]. After 4 weeks of intervention, supplementation with 5 mg/kg body weight/day of lycopene significantly suppressed high-cholesterol induced total and LDL cholesterol levels in serum. In addition, the amounts of cholesteryl ester in the aortae were significantly reduced in the animal group fed with lycopene. The extent of aortic surface lipid deposition was not influenced by lycopene.

## Materials and Methods

### Animal experiments

Male New Zealand White (NZW) rabbits from Charles River Laboratories (Sulzfeld, Germany) at 18 weeks (weight 3.3–4.1 kg) were used for this experiment. After an adaption period with standard diet, blood was collected after overnight fasting from the central ear artery to obtain basal lipid and lycopene levels. The animals were stratified into four groups (n = 9 animals each) based on blood lipid levels and body weights. The rabbits were housed in individual cages at 20°C under a 12-hour dark and light cycle. The control group was fed a standard diet (16.5% protein, 3.5% fat, 15.2% fibre, 7.2% ash, 19.3% starch, 6.5% sugar). The cholesterol group received the standard diet supplemented with 0.5% cholesterol (high-cholesterol diet, ssniff Spezialdiäten GmbH, Soest, Germany). The lycopene group received the high-cholesterol diet supplemented with 5 mg/kg body weight/day of lycopene in the form of lycopene beadlets mixed into the chow. The lycopene beadlets [redivivo™ (lycopene) 10% CWS/S-TG] were kindly provided by DSM Nutritional Products (Basel, Switzerland). The beadlets contain 10% lycopene in a cornstarch-coated matrix of modified starch and glucose with α-tocopherol (1.5%) and sodium ascorbate (5%) for stabilisation of lycopene. An additional animal group was fed a high-cholesterol diet with placebo beadlets (DSM Nutritional Products, Basel, Switzerland) that contain all the ingredients of lycopene beadlets except lycopene. All diets were pelletized under low pressure and low temperature to preserve the integrity of the beadlets. Both diets with beadlets were sealed under vacuum and stored with an oxygen adsorber in aliquots at −20°C until use. Constant lycopene content in the chow was verified during the experiment. Fresh food was provided every day and chow from the previous day was removed. Water and food were supplied ad libitum. Consumption of the diets was determined daily by weighing the chow. Body weights of the animals were recorded weekly and supply of the diets was adjusted accordingly. All diets were administered for 4 weeks. At the end of the study, the rabbits were anesthetized with pentobarbital sodium (20–40 mg/kg body weight intravenously) and blood was collected by heart puncture. Plasma and serum were obtained and frozen at −80°C for further analysis. Animals were sacrificed with 100 mg/kg body weight pentobarbital intravenously. Aortae were excised for assessment of atherosclerotic changes, vasorelaxation studies, and determinations of lipid levels. The animal study was carried out in strict accordance with national guidelines on the care and use of laboratory animals. The study was approved by the local ethics committee, the Landesamt für Gesundheit und Soziales (LAGeSo) Berlin, under the permit number G 007/08. All efforts were made to minimize suffering of animals.

### Lycopene plasma levels

Carotenoids were extracted as described [14], with slight modifications [15]. Echinenone (CaroteNature, Lupsingen, Switzerland) was used as internal standard. The measurements were carried out under subdued light to prevent photodegradation and isomerization. We employed a Merck L7200 autosampler, a Merck L7100 pump, a Merck L7450 PDA detector (Merck, Darmstadt, Germany), and a Jet Stream Plus column oven (Jasco, Groß-Umstadt, Germany) for determination of lycopene plasma levels by HPLC. We analysed 100 µl of plasma extracts on a C_30_ (250×4.6 mm, 5 µm) column (YMC, Dinslaken, Germany), preceded by a C_18_ ProntoSil 120-5-C18 H (10×4.0 mm, 5 µm) guard column (Bischoff, Leonberg, Germany) at 17±1°C with diode-array detection at 470 nm. The mobile phase (1.3 ml/min) consisted of methanol (MeOH) and methyl tert.-butyl ether (MTBE). We applied the following gradient procedure: (1) initial condition with 90% MeOH and 10% MTBE, (2) a 45-min linear gradient to 40% MeOH and 60% MTBE, (3) 40% MeOH and 60% MTBE for 15 min, (4) a 3-min linear gradient to 90% MeOH and 10% MTBE, (5) 90% MeOH and 10% MTBE for 7 min. Lycopene was quantified using (*all-E*)-lycopene reference material (CaroteNature, Lupsingen, Switzerland). The concentration of the lycopene stock solution was checked periodically in n-hexane at 472 nm by using its extinction coefficient E_(1%, 1 cm)_: 3450. The plasma lycopene levels are given as total lycopene: i.e., as the sum of all detected lycopene isomers.

### Measurement of serum blood lipids

Fasting total cholesterol, LDL cholesterol, HDL cholesterol and triglycerides in serum were determined using enzymatic methods with Cobas C701 module (Roche Diagnostics GmbH, Mannheim, Germany). Selective determination of HDL and LDL cholesterol utilizes the formation of water soluble complexes with LDL and dextran sulfate that are resistant to PEG-modified enzymes. Total-,LDL-, and HDL-cholesteryl esters were cleaved with cholesterol esterase to yield the respective free cholesterols. Triglycerides were hydrolyzed by lipoprotein lipase to obtain free glycerol. Free cholesterols were oxidised by cholesterol oxidase and glycerol by glycerol phosphate peroxidase. The amount of H_2_O_2_ generated during this oxidation process is proportional to the amount of cholesterol and glycerol that is subsequently converted by a colour reaction and measured photometrical. The following enzymatic assays were used: CHOD-PAP cobas® for total cholesterol, LDL-C plus second-generation cobas® for LDL cholesterol, HDL-C plus third-generation cobas® for HDL cholesterol, and GPO-PAP cobas® for triglycerides. The amounts of serum lipids for the respective lipid fractions were calculated according to the instructions of the manufacturer.

### Determination of initial aortic lesion area

The aortae were carefully dissected in situ, then excised and fixed with formalin. They were cut longitudinally and, after removal of formalin with destilled water and equilibration in 50% ethanol, the thoracic (2 cm above the diaphragm up to the Truncus brachiocephalicus) and abdominal (from diaphragm to 1 cm below the A. renalis) aortae were stained en face with Scarlet R (Sudan IV) for 5 minutes to visualise lipids. The aortae were subsequently rinsed with 70% ethanol and washed in distilled water. Stained aortae were photographed with a digital camera (Canon PowerShot S50). Total aortic area and Sudan IV-positive area was quantified using ImageJ version 1.36b software. The Sudan IV-positive area was calculated as percentage of the total aortic surface area. The analyst was blinded to the treatments.

### Lipid deposition in tissues

Lipid accumulation in left carotid arteries and in the ascending aorta was evaluated by Oil red O staining (Sudan Red 5B). Cryosections (5 µm thick) were mounted on slides, fixed in formalin, washed in distilled water, and equilibrated in 60% isopropyl ethanol. Slides were then stained in fresh Oil Red O solution for 10 minutes and differentiated in 60% isopropyl ethanol. After rinsing with destilled water, slides were stained with hematoxylin (for staining of nuclei) for 20 seconds, briefly rinsed with distilled water, blued for 10 minutes under water, and mounted in Aquatex. The percentage of Oil red O-stained area on the total surface area was calculated. The amounts of cholesteryl esters and other lipid subgroups in distal segments of the frozen thoracic aorta (1 cm above the diaphragm) were determined by electrospray ionisation tandem mass spectrometry (ESI-MS/MS) in positive-ion mode by use of a fragment ion of m/z 369, as described previously [16].

### Intima-media thickness

Histological evaluation was done by Elastica van Gieson staining. The ascending aorta was fixed in formalin and paraffin-embedded. Six consecutive sections, with an interval of 100 µm between sections, were analysed. Sections (2 µm thick) of the ascending aorta were stained with resorcin-fuchsin (for staining of elastic fibres) for 15 minutes, with hematoxylin (staining of nuclei) for 2 minutes, and picrofuchsin (staining of collagen and cytoplasm) for 10 minutes, followed by mounting in Entellan. Stained slides were visualised under a Leitz DM RBE (Leica, Wetzlar, Germany) light microscope and recorded with an AxioCam MRc microscope camera (Carl Zeiss MicroImaging, Göttingen, Germany). Images were analysed by a blinded analyst using the software AxioVision Rel 4.8.2 (Carl Zeiss MicroImaging, Göttingen, Germany). Mean intima-media thickness (in µm) is given for individual animals.

### Vasorelaxation studies

After 4 weeks of treatment, abdominal aortae (2 cm-sections below the parts that were used for en face) and right carotid arteries from the animals were rapidly excised, cleaned of surrounding tissue, and cut into rings of 2 mm length under semi-sterile conditions. The rings were then mounted on platinum hooks in 10 ml jacketed organ baths containing modified Krebs-Henseleit solution (composition in mM: 144 NaCl, 5.9 KCl, 1.6 CaCl_2_, 1.2 MgSO_4_, 1.2 KH_2_PO_4_, 25 NaHCO_3_, and 11.1 D-glucose) and 1 µM diclofenac. Tension was gradually adjusted to 2 g for 1 hour. The solution in the bath was kept at 37°C with a gas mixture of 5% CO_2_ and 95% O_2_. Following equilibration and submaximal pre-contraction with phenylephrine (1 µM for aortic rings and 0.5 µM for carotid rings), relaxation to increasing concentrations of the endothelium-dependent vasodilator acetylcholine (50 nM to 10 µM) was performed to obtain cumulative concentration-response curves. Maintenance of smooth-muscle integrity was confirmed by evaluation of endothelium-independent vasodilation to sodium nitroprusside (SNP; 1 nM to 1 µM).

### Markers of oxidative changes

Serum concentrations of monocyte chemoattractant protein-1 (MCP-1) as an inflammatory cardiovascular marker, were measured by enzyme-linked immunosorbent assays (ELISA; Cusabio Biotech, Wuhan, China). Lipid peroxidation products in serum were determined by spectrophotometric measurement of formation of thiobarbituric-acid reactive substances (TBARS), as described [17] with slight modifications. Levels of 8-iso-prostaglandin F_2α_ (8-iso-PGF_2α_; 8-isoprostane) as marker for oxidative stress were measured in plasma. Alkaline hydrolysis was performed to quantify the sum of free and lipid-esterified 8-iso-PGF_2α_. Prior to ELISA analysis, the plasma samples underwent affinity purification with an 8-isoprostane affinity purification kit (10368; Cayman Chemical, Ann Arbor, MI, USA). Total content of 8-isoprostane in plasma was measured by a specific 8-isoprostane enzyme-linked immunosorbent assay kit according to the instructions of the manufacturer (516351; Cayman Chemical, Ann Arbor, MI, USA).

### Statistical analysis

Values are expressed as mean ± SD unless otherwise indicated. Statistical analysis was performed with SigmaStat Version 3. Statistic calculations were carried out with One Way ANOVA on Ranks (Student-Newman-Keuls test) for multiple pairwise comparisons of groups when comparing medians and with Bonferroni t-test when comparing means. Level of significance was accepted at P≤0.05.

## Results

**Figure 1:**
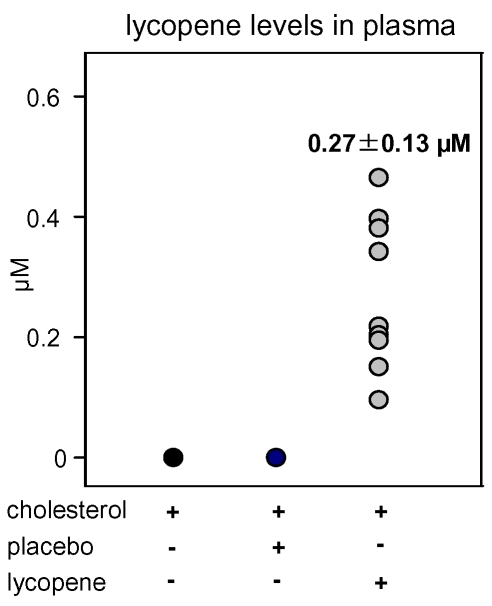
Plasma total lycopene levels in individual rabbits after feeding the indicated diets for 4 weeks. Cholesterol: animals were fed with a high-cholesterol diet (0.5% cholesterol); Placebo: high-cholesterol diet supplemented with placebo beadlets containing all ingredients of lycopene beadlets except lycopene; Lycopene: high-cholesterol diet supplemented with 5 mg lycopene/kg body weight/day as lycopene beadlets. The number in the graph represents the mean ± SD. n = 9 animals per group.

**Figure 2:**
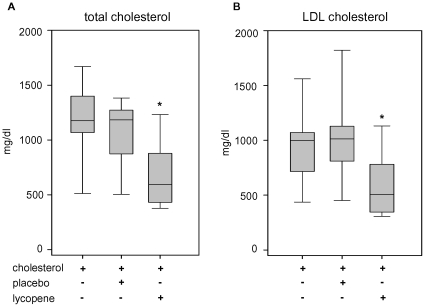
Blood lipid levels after intervention. Box plot of total cholesterol (**A**) and LDL cholesterol (**B**) levels in serum after supplementation with the indicated diets for 4 weeks. The boxes contain 50% of individual values; the middle line within the box represents the median. Total and LDL cholesterol levels were significantly lower in the lycopene group. *P<0.05 compared to cholesterol and placebo. n = 9 animals per group.

There were no significant differences in mean body weights and blood lipid levels between the animal groups at the beginning of the study. Within the high-cholesterol fed groups, no significant differences in uptake of the cholesterol diet were observed during 4 weeks of supplementation. At the end of the experiment, body weights of the rabbits did not significantly differ among any of the groups. Significantly elevated lycopene plasma levels were obtained in rabbits treated with lycopene for 4 weeks, whereas all other animal groups were devoid of lycopene ( Figure 1). Basal lipid levels in rabbits were very low (Table 1). The high-fat diet resulted in a rigorous increase in blood lipid concentrations in all high-cholesterol groups. However, serum lipid levels were substantially reduced after additional supplementation with lycopene. We observed a significant reduction to nearly 50% of the total cholesterol and LDL cholesterol serum levels in the lycopene group, in comparison to the high-cholesterol and the placebo groups (Figure 2). Concentrations of HDL cholesterol did not significantly differ between all high-cholesterol-fed groups. The LDL/HDL ratio significantly improved in the animals treated with lycopene, compared to placebo (Table 1).

**Table 1:** Serum lipid levels and LDL/HDL quotients.

	Total cholesterol	LDL cholesterol	HDL cholesterol	LDL/HDLquotient
Control	31±9	8±3	15±8	0.6±0.5
Cholesterol	1182±329	948±330	265±124	4.0±1.8
Placebo	1078±281	1020±372	246±88	4.3±1.1
Lycopene	664±286*	581±278*	229±97	2.7±1.2#

Values (in mg/dl) are mean ± SD from n = 9 animals per group.

*P<0.05 compared to cholesterol and placebo.

# P<0.05 compared to placebo.

**Figure 3:**
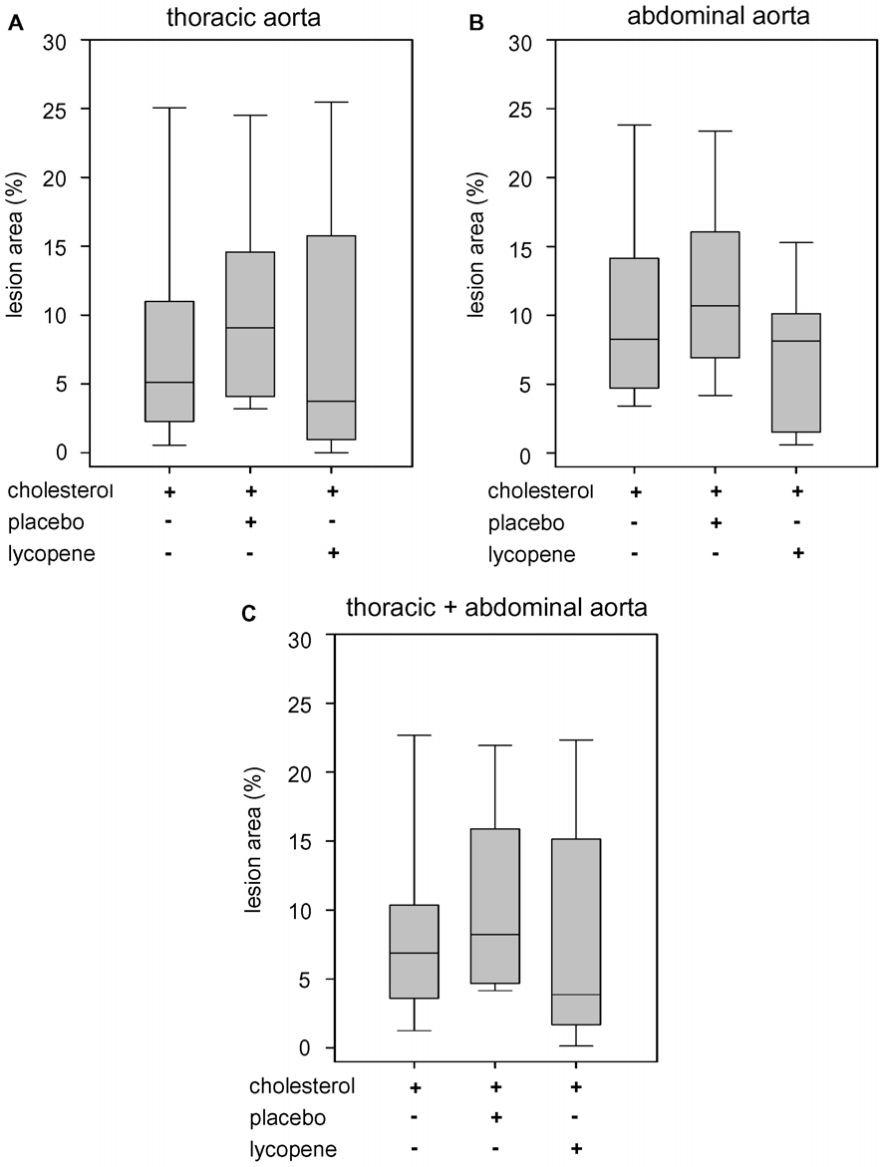
Surface lipid depositions in explanted aortae in the three high-cholesterol groups. Shown are box plots of the extent of lipid depositions in the thoracic aorta (**A**), in the abdominal aorta (**B**), and the sum of thoracic and abdominal aorta (**C**) as the percentage of lipid depositions compared to the total aortic surface area. The results were obtained after sudan IV-staining. n = 9 animals per group.

**Figure 4:**
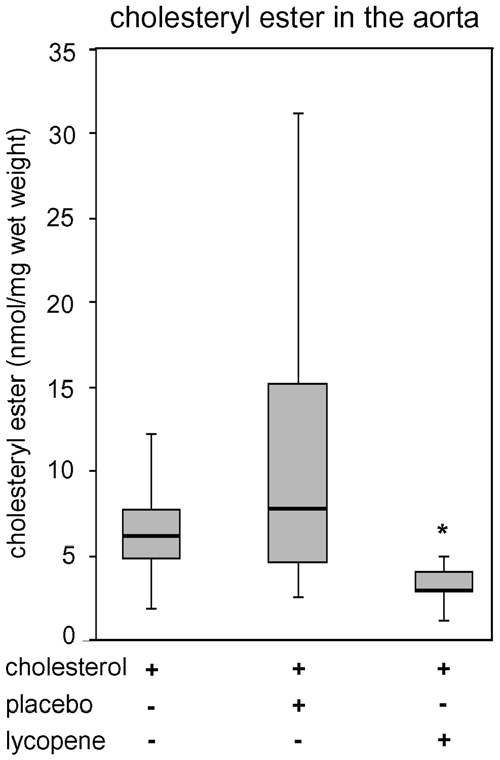
Levels of cholesteryl ester in the aortae of rabbits after supplementation for 4 weeks. Data are presented as box plots. The boxes contain 50% of individual values; the middle line within the box represents the median. The amount of cholesteryl ester was significantly lower in the lycopene group. *P<0.05 compared to cholesterol and placebo. n = 9 animals per group.

**Figure 5:**
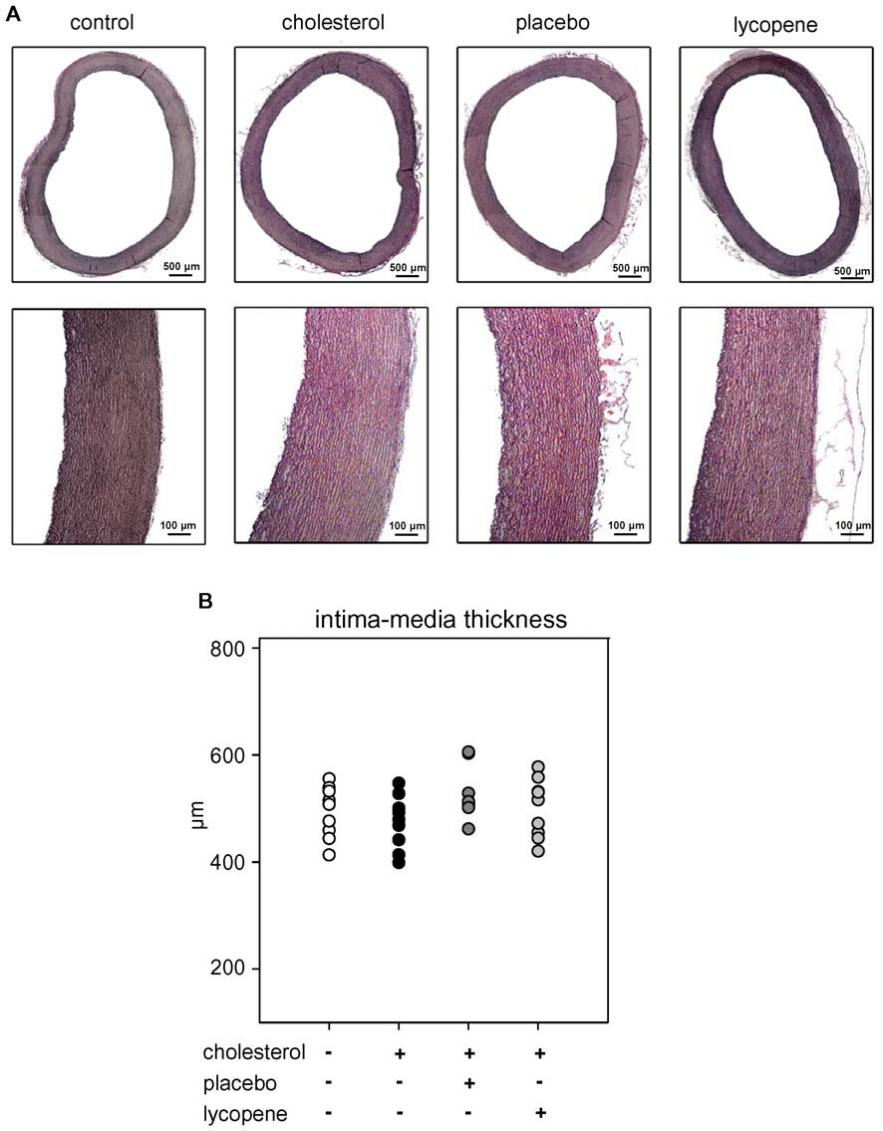
Microphotographs and intima-media thickness of the ascending aortae in individual animals after the indicated diets. (**A**) Representative microphotographs of Elastica van Gieson staining of the overall aortic cross-sections for each group (upper panel). Magnification of aortic sections (lower panel). (**B**) Mean intima-media thickness per animal in µm. Summary of all stained segments per animal from n = 9 animals per group.

Although lycopene strongly decreased serum cholesterol levels, the extent of surface lipid depositions in the aorta was not significantly reduced by lycopene. We found only a slight trend towards lower initial lesion area in the abdominal aorta, whereas no differences in the thoracic aorta were observed between all high-cholesterol fed animals (Figure 3). The control group receiving a standard diet did not show any lipid depositions (data not shown). In contrast, the amounts of cholesteryl ester within the aorta that were increased after high-cholesterol diet were significantly reduced in the lycopene group (Figure 4). Levels of phosphatidylcholine (PC), lysophosphatidylcholine (LPC), phosphatidylglycerol (PG), phosphatidylserine (PS), sphingomyelin (SPM), and free cholesterol (FC) did not differ between treatment groups (data not shown). No pronounced aortic lesions (Figure 5A) and no significant increase in aortic intima-media thickness were observed after high-cholesterol diet (Figure 5B), which illustrates a pre-atherosclerotic state with the absence of atherosclerotic changes in the aortae. In addition, Oil red O staining revealed no lipid deposition in the carotid arteries after 4 weeks of high-cholesterol diet. Accumulation of lipids within the aortic root was negligible, and no differences between all high-cholesterol fed animal groups were detected (data not shown).

**Figure 6:**
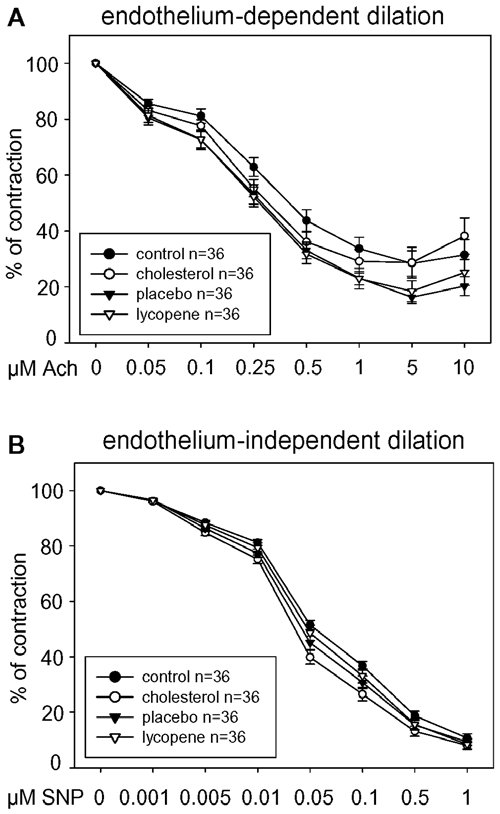
In vitro vasorelaxation studies after the animal experiment. Endothelial-dependent vasodilation induced by acetylcholine (Ach, **A**) and endothelium-independent vasodilation by sodium nitroprusside (SNP, **B**) in isolated rabbit aortic rings after treatments with the indicated diets for 4 weeks. Graphs show relaxation as percent of maximal contraction. Data are mean ± SEM from n = 9 animals per group with 4 aortic rings per animal.

Endothelial-dependent and endothelial-independent vasodilation in isolated rabbit aortic rings was measured after 4 weeks of diets. No differences were found between all animal groups (Figure 6). Similar results were obtained with isolated rabbit carotid rings (data not shown). In accordance with the results of the vasorelaxation experiments, plasma cGMP levels were not influenced by the high-cholesterol diet and lycopene had no impact (data not shown). Also, signs of pro-oxidative changes such as serum levels of monocyte chemoattractant protein-1 (MCP-1) and lipid peroxidation (TBARS) in serum as well as plasma levels of 8-isoprostane (8-iso-prostaglandin F_2α_) were not increased after the high-cholesterol diet and were not influenced by lycopene supplementation (data not shown).

## Discussion

The main results of our study are that lycopene supplementation for 4 weeks strongly suppressed diet-induced increases in total and LDL cholesterol levels in serum and reduced the accumulation of cholesteryl esters in aortic tissue of rabbits. However, we observed no significant differences in surface lipid depositions among all high-cholesterol fed groups. Furthermore, no advanced atherosclerotic changes in the aortae, increase of pro-oxidant parameters in serum, or impairments of vasoreactivity in isolated blood vessels were detected in all animal groups. The latter findings indicate that the administration of the high-cholesterol diet for 4 weeks resulted in a very early, initial state of atherosclerosis in our rabbit model.

High LDL cholesterol levels represent an independent risk factor for the development and progression of cardiovascular diseases. For every reduction in 25 mg/dl of LDL cholesterol, the risk of vascular mortality was reduced by 11% and of major coronary events, by 16% [18]. A high LDL/HDL ratio is predictive for adverse cardiovascular events [19], [20]. Although lycopene did not increase HDL cholesterol levels in our study, the LDL/HDL quotient was improved in comparison to the high-cholesterol and the placebo groups, further emphasizing a beneficial cardiovascular profile of lycopene. Similar reductions of blood lipid levels by lycopene had been observed in two other rabbit experiments. High-fat induced hyperlipidemia was significantly suppressed by various doses of lycopene (4 and 8 mg/kg body weight/day) in New Zealand White rabbits after treatments for 4 and 8 weeks. However, a slight increase in HDL cholesterol levels did not reach statistical significance [21]. In the same animal model of atherosclerosis, supplementation with three doses of lycopene in the chow for 12 weeks dose-dependently decreased diet-induced serum total cholesterol and LDL cholesterol levels and increased HDL cholesterol [22]. In contrast, supplementation with a lycopene-enriched tomato extract (15 mg/kg body weight/day lycopene) in the diet for 16 weeks had no effect on plasma cholesterol levels in Watanabe Heritable Hyperlipidemic (WWHL) rabbits [23]. The reason for the lack of effects of lycopene in WWHL rabbits may originate in defective LDL receptors in these animals [24]. Since lycopene is transported in the blood mainly in LDL particles [25], a functional LDL receptor may be essential to obtain cardiovascular beneficial effects of lycopene.

What are the mechanisms behind the cholesterol-lowering effects of lycopene? It should be noticed in this context that basal cholesterol levels in rabbits are very low. Primarily, lycopene led to suppressed cholesterol uptake rather than to a reduction of existing circulating cholesterol levels in these animals. In human macrophages, lycopene reduced intracellular cholesterol levels by decreasing the expression of 3-hydroxy-3-methyl glutaryl Coenzyme A (HMG-CoA) reductase, a rate-limiting enzyme in cholesterol biosynthesis [26]. However, expression levels of HMG-CoA reductase in the liver were not affected by supplementation with lycopene in our animal experiment (data not shown). Increased faecal cholesterol excretion, together with reduced liver HMG-CoA reductase activity, was shown after dietary lycopene intake in rabbits, suggesting decreased intestinal cholesterol absorption and biosynthesis [22]. Results for the effects of lycopene on plasma lipid levels in human intervention studies are not consistent. Supplementation with tomato extract capsules (4 mg lycopene) daily for 6 months decreased total cholesterol and LDL cholesterol levels in postmenopausal women [27]. No effects on blood lipid levels were obtained after supplementation with a tomato extract containing 15 mg lycopene daily for 8 weeks in mild hypertensive patients [28]. A recent meta-analysis of human intervention trials revealed significant reduction in total and LDL cholesterol only at doses of ≥25 mg lycopene daily. Doses of <25 mg lycopene had no effect on cholesterol serum levels. HDL cholesterol was not changed by lycopene uptake, independently of dosages applied [12].

Despite markedly reduced serum cholesterol levels, we found no effects on surface lipid deposition in the aortae of lycopene-supplemented rabbits. This represents an unexpected result. In the studies with atherosclerotic NZW rabbits noted above, the decrease in blood lipid levels was associated with a concomitant reduction in aortic plaque area in lycopene-treated animals [21], [22]. In one study, the lipid-lowering effects and the extent of atherosclerotic lesion reduction by lycopene were comparable with statins [21]. However, there are a number of differences in study design between these studies und ours. Changes in aortic lesion size were assessed after 8 and 12 weeks, whereas in our study we aimed at shorter intervention times. Hu et al. administered the lycopene intragastrically by catheter inserted into the stomach, and the high-fat diet consisted of 1% cholesterol, whereas we used 0.5% cholesterol in our study. In addition to determining cholesterol-lowering effects in plasma, we found that supplementation with lycopene reduced the amount of cholesteryl ester in the aorta. This represents a novel and relevant finding. The invasion of lipids in the aortic tissue is the initial step in plaque progression and represents a prerequisite for development of advanced aortic plaques. The reduced accumulation of lipids within the aortic tissue, as opposed to equal surface deposition, could point to a beneficial effect of lycopene on further progression of atherosclerotic changes.

Several reasons could potentially explain why we did not observe reduced aortic surface lipid deposition, in addition to decreased accumulation of lipids within the aortae. It could be argued that the lycopene plasma levels obtained in our study were too low to exert anti-atherogenic effects beyond reduced tissue lipid levels. The dosage of 5 mg/kg body weight/day of lycopene for 4 weeks resulted in significantly increased total lycopene plasma levels (0.27 µM) in our study. This concentration was even higher in comparison to the study of Hu et al. describing reduced atherosclerotic lesions in rabbits after supplementation with lycopene [21]. Another difference of our study represents the duration of high-cholesterol diet administration. Certainly, longer intervention times (as 4 weeks used in our study) would result in more pronounced atherosclerotic lesions. However, our major objective was to investigate the effects of lycopene on cardiovascular parameters before the establishment of distinct atherosclerotic lesions in the vessels. We therefore decided to conduct our study for a shorter time period of 4 weeks. We observed very early (not advanced) signs of atherosclerosis in our animals. These initial states of atherosclerosis were influenced by lycopene supplementation. However, in contrast to other studies with longer duration [29], we did not observe diminished endothelial-dependent vasodilation in aortic rings after 4 weeks of high-cholesterol diet, suggesting that longer intervention times may have resulted in different outcomes.

There are, to date, no prospective human intervention studies on the effects of lycopene or tomato products on the progression of atherosclerosis. An inverse correlation of plasma lycopene levels to cardiovascular events was found in various epidemiological studies [30], [31]. Therefore, it needs to be proven whether supplementation with lycopene is able to delay the onset of cardiovascular diseases in humans.

In conclusion, increased lycopene plasma levels after supplementation were associated with reduced total and LDL cholesterol in serum as well as with lower aortic cholesteryl ester levels in New Zealand White (NZW) rabbits. However, no significant impact of lycopene on surface aortic lipid deposition was observed. It is accordingly still eligible to ascertain the consequences of reduced blood cholesterol levels by lycopene on the progression of cardiovascular diseases in humans.
